# New index of organic mass enrichment in sea spray aerosols linked with senescent status in marine phytoplankton

**DOI:** 10.1038/s41598-020-73718-5

**Published:** 2020-10-12

**Authors:** Yuzo Miyazaki, Koji Suzuki, Eri Tachibana, Youhei Yamashita, Astrid Müller, Kaori Kawana, Jun Nishioka

**Affiliations:** 1 Institute of Low Temperature Science, Hokkaido University, Sapporo, Japan; 2 Faculty of Environmental Earth Science, Hokkaido University, Sapporo, Japan; 3 Graduate School of Environmental Science, Hokkaido University, Sapporo, Japan; 4 Present Address: National Institute for Environmental Studies, Tsukuba, Japan; 5 Graduate School of Environmental Studies, Nagoya University, Nagoya, Japan; 6 Present Address: Japan Agency for Marine-Earth Science and Technology, Yokohama, Japan

**Keywords:** Biogeochemistry, Atmospheric science, Biogeochemistry

## Abstract

Linking the amount of organic matter (OM) in sea spray aerosols (SSAs) to biological processes in ocean surface is essential for understanding marine aerosol formation and their potential to affect cloud formation. To date, chlorophyll (Chl) *a* concentration has been widely used as a surrogate for surface phytoplankton biomass or productivity to predict the relative abundance of OM in SSAs (OM_SSA_). Here we show a new index to present OM_SSA_ using concentrations of Chl *a* and chlorophyllide (Chllide) *a*, which is a breakdown product of Chl *a* and has been used as a biomarker of senescent algal cells. The index was compared with submicrometer OM_SSA_, based on surface seawater and aerosol samples obtained during the pre-bloom in the western subarctic Pacific. Our results showed that the OM_SSA_ was highly correlated with this unique index, suggesting that the OM_SSA_ was closely linked with senescent algal cells and/or cell lysis. Furthermore, the hygroscopicity parameters *κ* derived from water-extracted SSA samples implied a reduction in the SSA hygroscopicity with increasing senescent status of phytoplankton. The index can represent OM_SSA_ on a timescale of a day during the pre-bloom period, which should be further examined over different oceanic regions.

## Introduction

Marine atmospheric aerosols act as cloud condensation nuclei (CCN) and ice nuclei (IN), controlling the atmospheric radiative budget through cloud formation^1^, and thus play a vital role in the climate system. The ocean surface serves as a significant source of aerosols in terms of both particle number and mass concentrations^2^. In particular, ocean-derived aerosol particles contain a complex mixture of organic matter (OM), which has potential to affect the hygroscopic property and CCN activity of aerosols^3–5^. One of the largest global sources of directly emitted (primary) aerosols is wind-driven particle production at the ocean surface^6,7^. Assessing the ocean-to-cloud relationship requires knowledge of the marine biogeochemical processes that produce OM in ocean surface waters, followed by emissions of OM from the ocean and subsequent atmospheric aerosol formation, and the transition of a subset of the aerosol population to cloud droplets and ice crystals.

Chlorophyll *a* (Chl *a*) concentration in seawater has been widely used as a proxy for phytoplankton biomass or productivity^7,8^. Global modeling studies have also used Chl *a* to estimate the organic fraction of sea spray aerosol (SSA)^9–11^. This is due to the wide spatiotemporal coverage of satellite-derived Chl *a* measurements over the oceans. Long et al.^9^ reported that linear source functions based on Chl *a* underpredicted organic carbon (OC) enrichment for nascent SSA produced from oligotrophic waters, whereas those overestimated the OC enrichment from highly productive waters.

Primary organic aerosols may originate from short-lived, transient pools of labile OM in surface seawater associated with phytoplankton blooms, combined with the relatively constant background of refractory dissolved OM (DOM) in the ocean^12^. Quinn et al.^13^ found that instantaneous Chl *a* and OM content in SSAs showed almost no relationship with the presence or absence of plankton bloom both in the North Atlantic Ocean and California coastal waters. They pointed out that the OC fraction in freshly emitted SSA was effectively invariant at 5%, regardless of the variability of Chl *a* levels in seawater. Quinn et al.^13^ concluded that a large OC reservoir existing in surface seawater results in the enrichment of OM in SSA, which could be irrelevant to the concentration of Chl *a* in seawater over a variety of oceanic regions.

Rinaldi et al.^8^ showed evidence of a systematic delay of 8–10 days between a time series of Chl *a* and OM in SSA, which was attributable to the timescale of the biological processes responsible for the production of transferable organic materials during the bloom evolution. Regarding the time lag between Chl *a* concentration and OM enrichment in aerosols, O’Dowd et al.^14^ suggested that the production of OM in SSA is likely linked to the decay of phytoplankton bloom and cell lysis rather than their biological activity—the waning of prokaryotes (bacteria and archaea) and phytoplankton due to viral infections after the waxing stage. An investigation is still needed to understand the factors controlling the OM enrichment in SSA in different bloom stages.

The Oyashio is a wind-driven western boundary current flowing southwestward of the western subarctic gyre of the North Pacific^15^. In the Oyashio and its coastal region, phytoplankton bloom occurs from March to June^16,17^. This oceanic region in spring has potential for enhanced concentration of OM at the sea surface^18^, providing an opportunity to investigate the chemical and biological linkages of OM at the ocean–atmosphere interface. This study presents a new application of a proxy for OM mass-fraction enrichment in SSA on a daily scale, based on a shipboard measurement of ambient aerosols and surface seawater in the western subarctic Pacific. The measurements were made during the pre-bloom, when the amount of OM in surface seawater is expected to be sensitive to marine biological activity (Supplementary Figure S1). An index using concentrations of Chlorophyllide (Chllide) *a*, a breakdown product of Chl *a* by the enzyme chlorophyllase, provides the relative abundance of senescent algal cells to the total phytoplankton. We discuss the validity of this index by comparing it with organic enrichment in SSA, which is defined by stable carbon isotope ratio and local wind data, based on the cruise measurements of ambient aerosol and surface seawater.

## Results and discussion

### Chemical properties of submicrometer aerosols

**Figure 1:**
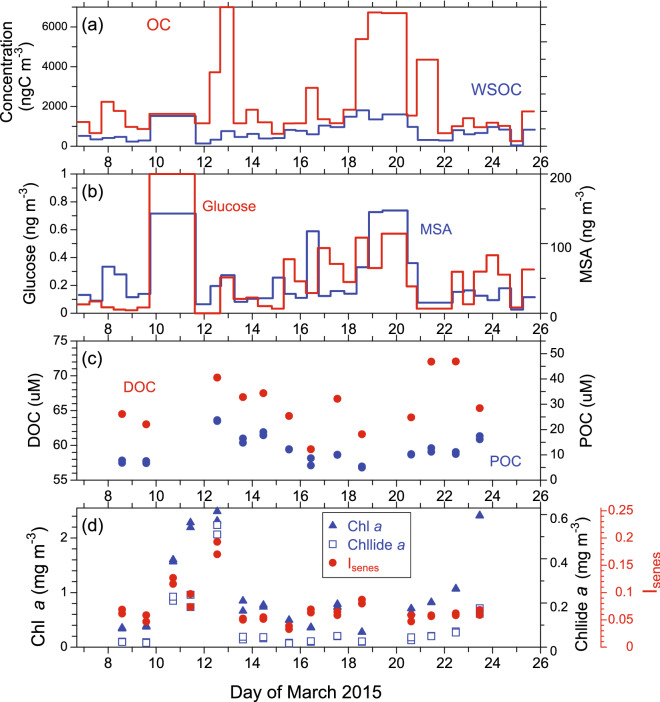
Time series of the concentrations of (**a**) organic carbon (OC) and water-soluble organic carbon (WSOC), (**b**) glucose and MSA in the submicrometer aerosols, (**c**) DOC and POC, and (**d**) the surface seawater chlorophyll (Chl) *a* and chlorophyllide (Chllide) *a* concentrations with I_senes_ [= Chllride *a* / (Chllride *a* + Chl *a*)] along the cruise track during the KH-15–1 cruise in the Oyashio and its coastal regions in the western subarctic Pacific in 2015.

Figure 1a presents the time series of the mass concentrations of OC and water-soluble OC (WSOC) in the submicrometer aerosol particles during the cruise. Overall, the patterns of the temporal OC and WSOC variations were similar to each other (*r* = 0.632, *p* < 0.001), showing greater variability throughout the entire cruise. The concentrations of OC exceeded 3000 ngC m^−3^ in some cases. The average concentrations of OC and WSOC were 2278 ± 2067 ngC m^−3^ and 717 ± 440 ngC m^−3^, respectively. These concentration levels are 2–3 times higher than those in submicrometer particles observed during the post-bloom period in the western subarctic Pacific^19,20^, likely because the current study region includes the coastal region.

Temporal variations of glucose and methanesulfonic acid (MSA) in the submicrometer aerosols are shown in Fig. 1b. Glucose is used here as a tracer for primary marine aerosols, as SSAs contain a substantial amount of monosaccharides, including glucose^21,22^. MSA is considered to be either produced by gas-phase MSA directly scavenged by aerosols or rapidly produced in the aqueous phase from scavenged dimethylsulfoxide (DMSO) and methanesulfinic acid (MSIA)^23^, particularly under conditions with high relative humidity typical of the marine boundary layer (MBL). Throughout the cruise, the temporal variations of glucose and MSA were generally similar to those of OC and WSOC on a timescale of a few days. This similarity in the temporal trends of the mass concentrations suggests that the observed OC and WSOC in the submicrometer aerosols were significantly affected by marine biogenic sources. It is noteworthy that the timing of increases in the concentrations of glucose relative to those of MSA sometimes differed on a half-day to a day scale, likely due to the different contributions of primary and secondary sources to the observed aerosol mass in each aerosol sample.

### Chemical and biological properties of surface seawater

Figure 1c shows the concentrations of dissolved OC (DOC) and particulate OC (POC) in surface seawater at each sampling station corresponding to the aerosol sampling locations. Surface seawater DOC is defined as organic matter that penetrates through a 0.22-μm Durapore filter and contains particulate matter including viruses and colloids^24^. DOC concentrations during the cruise varied within a narrow range (59.4–72.1 μM C), with an average of 65.9 ± 3.7 μM C. During the same expedition, fluorescence intensities of protein-like component in DOC determined by excitation-emission matrix and parallel factor analysis showed the higher level in the sea surface, whose spatial distribution was similar to that of Chl *a*^24^. POC in surface seawater is operationally defined here as organic matter retained on a pre-combusted Whatman GF/F glass-fiber filter (nominal pore size of 0.7 μm)^18^, which contains a mixture of living planktonic organisms and detritus. POC concentrations in the surface seawater samples ranged from 5.0 to 23.7 μM C with an average of 12.0 ± 5.2 μM C. On average, DOC dominated TOC (= POC + DOC), which accounted for 85 ± 5% of TOC during the cruise.

Chl *a* concentrations ranged from 0.27 to 2.49 mg m^−3^ with an average of 0.88 ± 0.70 mg m^−3^ in this study. Concentrations of Chl *a* and POC in surface seawater showed a significant positive correlation (R^2^ = 0.60) during the sampling period (Fig. 1c,d). At all the seawater sampling stations, diatoms were predominant, accounting for 54–96% of the total Chl *a*, which was determined by multiple regression analysis based on diagnostic pigment signatures^25^. Furthermore, Yoshida et al.^25^ reported that *Thalassiosira* generally dominated the diatom assemblages in seawater during the cruise. Chllide *a* is a breakdown product of Chl *a* by the enzyme chlorophyllase, which has been used as a biomarker for senescent algal cells, particularly in diatoms^17,26^. For the bulk surface seawater in this study, the Chllide *a* concentrations ranged from 0.02 to 0.55 mg m^−3^ with an average of 0.09 ± 0.14 mg m^−3^, an order of magnitude lower than those of Chl *a* (Fig. 1d). The average concentration ratio of Chllide *a*/Chl *a* (9.6%) was well within the range (< 12%) in the pre-bloom phase reported in a similar oceanic region^17^. It should be noted that even under pre-bloom conditions, senescent phytoplankton cells could occur, because resource (light and/or nutrient) requirements differ among species (e.g., Sarthou et al.^27^), and lower temperatures reduced the photosynthetic capability of phytoplankton in coastal Oyashio waters during the cruise^25^. Although the fraction of Chllide *a* relative to Chl *a* was small, the current results clearly suggest that senescent cells existed even under the pre-bloom condition.

Here, we used an indicator (I_senes_) for the relative abundance of senescent algal cells to the total phytoplankton cells, which is defined as follows:

1 $${\text{I}}_{{{\text{senses}}}} = [{\text{Chllide}}\;a]{/}([{\text{Chllide}}\;a] + [{\text{Chl}}\;a])$$

where [Chl *a*] and [Chllide *a*] represent the concentrations of Chl *a* and Chllide *a* in the surface seawater, respectively^17^. Suzuki et al.^17^ showed that an index of diatom bloom development is positively correlated with I_senes_ regardless of the oceanic region in the western subarctic Pacific, demonstrating the validity of this I_senes_. In the present study, variations of I_senes_ were generally similar to those of Chl *a*, but in some cases, these parameters showed an opposite trend on a timescale of a day (e.g., Mar. 11, 16, 18, and 23; Fig. 1d).

**Figure 2:**
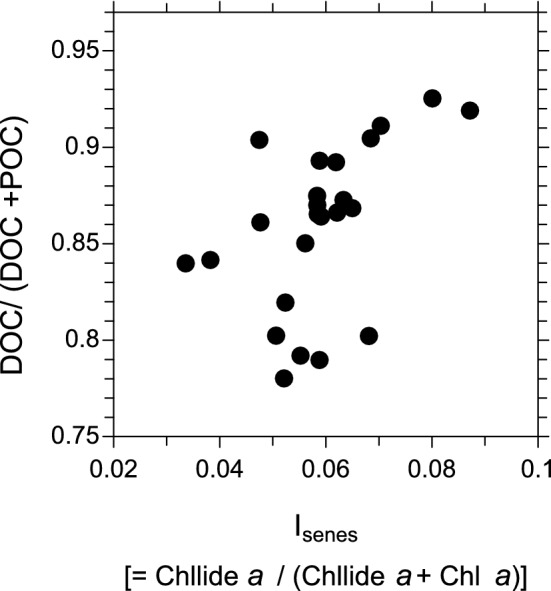
The concentration ratios of DOC/TOC (μM C/μM C) as a function of I_senes_ in the surface seawater samples during the cruise. Here TOC = DOC + POC.

Figure 2 presents the relationship between the DOC/TOC ratio and I_senes_ in the surface seawater samples obtained during the entire cruise. In general, the DOC/TOC tended to increase with increasing I_senes_ values. This relationship supports a possible coupling between DOC and senescent status in marine phytoplankton. The relative increase in DOC with increasing I_senes_ is partly attributable to an increase in extracellular organic matter produced by senescent phytoplankton. Indeed, Nosaka et al.^18^ observed higher ratios of DOC/TOC produced by phytoplankton during the post-bloom phase in the Oyashio waters of the western North Pacific. They suggested that the excretion of DOC, such as acid polysaccharides by diatoms, can produce transparent exopolymer particles (TEP) in seawater over this oceanic region.

### Organic mass enrichment in sea spray aerosols versus a biomarker for senescent algal cells

To investigate OM enrichment in SSA for senescent status in sea surface phytoplankton, submicrometer aerosol data representing SSA characteristics need to be selected from the ambient aerosol measurements. Characterization of SSAs in ambient air requires sampling and analytical methods that isolate these particles from being affected by preexisting or transported ambient gases and particles. A method using stable carbon isotope ratios (δ^13^C) combined with molecular markers provides robust tools to determine the contributions of marine and terrestrial sources to OC in marine aerosols^22,28–31^. Miyazaki et al.^31^ specifically selected aerosol data with characteristics of SSA observed during the same cruise as in this study. Their method for the selection of SSA samples was based on the δ^13^C of TC (δ^13^C_TC_) and water-soluble OC (WSOC; δ^13^C_WSOC_) together with local wind speeds. They showed a significant positive correlation between WSOC and glucose in the SSA samples, supporting the SSA’s nascent characteristics. The SSA sample is defined here as that showing the δ^13^C_TC_ and δ^13^C_WSOC_ values higher than − 22‰ under conditions of local wind speeds of > 5 m s^−1^. In this study, six samples were designate as SSA samples. For the selected SSA samples, all δ^13^C_TC_ values were within the values of marine sources with a negligible contribution of elemental carbon (EC; < 0.02 μg C m^−3^, see Fig. 1 of Miyazaki et al.^31^). Therefore, δ^13^C_TC_ can be assumed here to represent the δ^13^C value of OC. It is noted that seawater temperature may possibly affect the δ^13^C values in aerosols due to the isotope equilibrium fractionation linked to photosynthesis of phytoplankton^32^. However, the relation between the δ^13^C in SSA samples and surface sea temperature (SST) was not distinctly observed during aerosol sampling, which is partly attributed to the small variation in the average SST (~ 0.6–1.4 °C) during SSA sampling in this study^31^.

**Figure 3:**
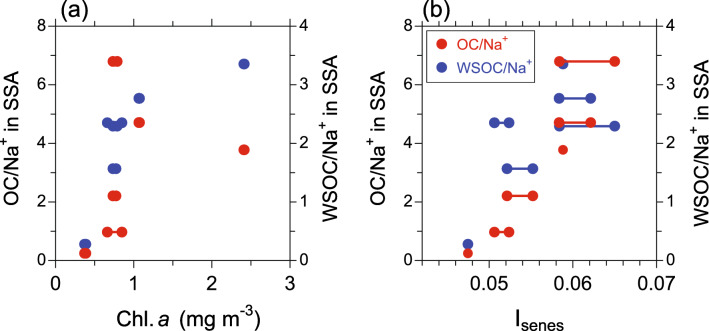
The OC/Na^+^ and WSOC/Na^+^ ratios in the SSA samples as functions of (**a**) Chl *a* concentration and (**b**) I_senes_ in surface seawater (SSW). For each one SSA sample, one or two corresponding measurement data of Chl *a* and Chllide *a* in SSW were obtained, so that the number of the data point in the panels is more than six of the SSA samples. The individual SSW data points corresponding to the identical aerosol sampling data are connected with a straight line.

To explore the correlation between OM enrichment in the SSA samples and phytoplankton biomass in terms of Chl *a* or their senescent status, Fig. 3 presents the OC/Na^+^ and WSOC/Na^+^ ratios in the SSA as functions of the Chl *a* concentrations and I_senes_. The term “enrichment” here refers to the amount of OC relative to Na^+^ in the SSA samples in this study. For the 12-h aerosol sampling, the OC/Na^+^ and WSOC/Na^+^ generally showed larger values with increasing the Chl *a* concentrations, although their correlations were not evident (*r* < 0.306, *p* > 0.05; Fig. 3a). The results were in agreement with the previous study that showed a correlation coefficient between satellite-derived Chl *a* and OM enrichment in sea salt particles on a timescale of a day, was lower than those on a weekly and monthly time scale^14^. In contrast, it is apparent that the OC/Na^+^ and WSOC/Na^+^ ratios showed a significant positive relationship with I_senes_ with *r* of 0.768 (*p* < 0.01) and 0.721 (*p* < 0.01), respectively (Fig. 3b). This result suggests that the OM enrichment in SSA is closely linked with the abundance of senescent algal cells relative to the total phytoplankton cells on a timescale of a day. Our results also support the concept that the production of OM in SSA is likely linked to senescent algal cells and/or cell lysis. It is noted that the OC/Na^+^ ratios observed in this study were generally larger than the typical range (0.1–2.0) previously reported for submicrometer marine primary OA^33^. This is probably because the ratio in the coastal region as observed in this study is generally larger than that in the open ocean^21^.

Previous studies suggest that OM enrichment in SSA is driven by insoluble colloids and nanogels^34^, forming surface-active organic materials. These organic materials are sufficiently small for inclusion as the operationally defined DOC, which contains viruses and cellular fragments owing to cell death or cell burst induced by a viral infection. Gel-like particles are composed of macromolecules and colloids. These organic materials are produced from surface-active polysaccharides and proteinaceous matter, which are ubiquitous at the ocean surface. In the present study, the observed OM in SSA, particularly WSOC, showed a significant correlation with glucose (*r* = 0.931, *p* < 0.001), a decomposed product of polysaccharides. The current results indicate that senescent status is particularly sensitive to OM enrichment in SSA during the pre-bloom phase on a daily timescale.

### Hygroscopicity parameters of sea spray aerosols and senescent status in marine phytoplankton

**Figure 4:**
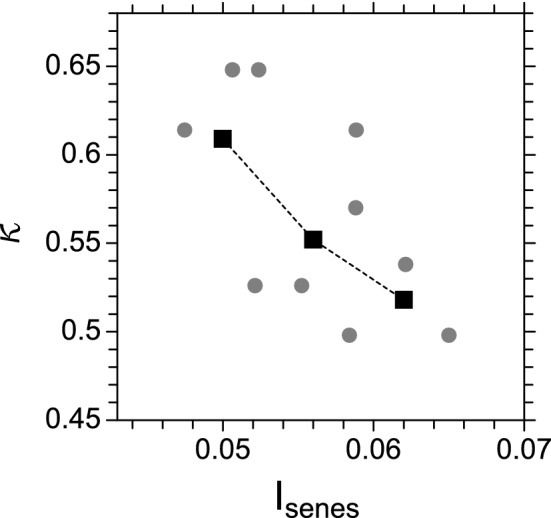
Hygroscopicity parameter *κ* in the SSA samples as a function of the corresponding I_senes_ in surface seawater. A gray solid circle indicate the individual data, whereas a black solid square indicates the average *κ* value in each I_senes_ bin of 0.047–0.053, 0.053–0.059, and 0.059–0.065.

With the water-extracted SSA samples defined here, the CCN activity was measured as the hygroscopicity parameter *κ* by nebulization of the filter extracts. Figure 4 displays *κ* in the SSA samples as a function of the corresponding I_senes_ in surface seawater. The κ values ranged between 0.50 and 0.65, with an overall average of 0.57 ± 0.06. These *κ* values are at the upper end of the range of values for ambient marine aerosol particles obtained by in-situ field measurements in high latitude oceanic areas (typically 0.1–0.6)^35,36^. Given that the aerosol from the filter extracts is completely water-soluble, this is to be expected. Although the number of available data is limited, the *κ* value tends to be smaller with increasing I_senes_ (Fig. 4). This implies that the reduction of hygroscopicity in SSA is associated with the aging process of phytoplankton cells and the subsequent increase of OM in SSA.

The current result is similar to previous studies showing a decrease in the hygroscopicity parameter by 9–37% when phytoplankton exudates or DOC-rich algae cultures were added to seawater in a laboratory experiment^37^. Furthermore, in that previous experiment, CCN activity has been found to depend on the type of algal exudate using a seawater proxy^37^, where the experiment with diatomaceous exudate showed more significant effects on the critical supersaturation than the experiments with nanoplankton exudate. Fuentes et al.^37^ found that the reduction in CCN activity was taxon-specific, with the DOM released by diatoms causing a greater reduction in CCN activity than that released by prymnesiophytes. Specifically, independent cultures of *Thalassiosira Rotula*, *Chaetoceros* sp., *Emiliania Huxleyi*, and *Phaeocystis* cf. *Globosa* in artificial seawater showed *κ* values of 0.95–1.21, which were lower than those (1.3–1.5) for the artificial seawater alone in their study. In wave channel experiments involving the heterotrophic bacterium *Alteromonas* spp. and the green microalga *Dunaliella Tertiolecta*, Collins et al.^38^ reported *κ* values as low as 0.12. In the current study, *κ* of OM (κ_OM_) for the SSA samples was calculated to be 0.44 ± 0.12 on the basis of the chemical fraction in the SSA samples (see methods section). The *κ* values shown in this study are within a range of the above values derived by the laboratory experiments. This study points to a reduction in the SSA hygroscopicity with increasing senescent status of diatom, suggested by the field measurements for the first time.

To summarize, the current study provides a new application of the index using concentrations of Chllide *a*, which is a biomarker for senescent algal cells (particularly diatoms), combined with Chl *a* concentration to represent the amount of OM in SSAs in the pre-bloom phase. This index should be further examined if it applies to the different stages of the bloom, namely phytoplankton bloom and post-bloom period, in future studies. Most bloom-forming diatoms show the chorophyllase activity converting from Chl *a* to Chllide *a*, whereas some algal groups such as the dinoflagellate *Gymnodinium* species, which sometimes bloom in coastal waters, do not possess the enzyme activity^39,40^. Therefore, the algal senescent index would be particularly useful during diatom bloom. Because diatoms are distributed worldwide in surface waters^41^, the new index has the potential to be a robust marker of the OM amount in SSA.

It should be noted that satellite remote sensing cannot distinguish Chllide *a* from Chl *a*, because optical properties of these two pigments are almost identical^42^. Instead, field data of Chllide *a* and Chl *a* concentrations over a variety of oceanic regions are publicly available (e.g., NASA bio-Optical Marine Algorithm Dataset (NOMAD)^43^, https://seabass.gsfc.nasa.gov/wiki/NOMAD). These datasets can be implemented in the climate models. In order to develop realistic parametrizations of the organic enrichment in SSA and subsequent emission to the atmosphere, further measurements of surface seawater and nascent SSA properties are required to confirm the results presented here.

## Methods

### Ambient aerosol sample collection

Ambient aerosol samples were collected from March 6 to 25, 2015, on board the R/V *Hakuho Maru* (JAMSTEC/the Univ. of Tokyo). Sampling was conducted during cruise the KH-15-1 expedition in the Oyashio and its coastal regions in the western North Pacific^24,25,31^ (Fig. S1). The aerosol samples were collected continuously using a high-volume air sampler (HVAS; Model 120SL, Kimoto Electric, Osaka, Japan) on the deck above the bridge of the ship. A cascade impactor (CI; Model TE-234, Tisch Environmental, Cleves, OH, USA) attached to the HVAS was used to collect size-segregated particles^31^ at a flow rate of 1130 L min^−1^ without temperature and humidity control. Here, we used analytical results from the bottom stage of the CI, which collected particles with an aerodynamic diameter (D_p_) < 0.95 μm. These particles are referred to as submicrometer aerosol particles in this study. The submicrometer aerosol sampling was made during local daytime and nighttime with a sampling duration of approximately 12–24 h. The samples were collected on quartz fiber filters (25 cm × 20 cm), pre-combusted at 410 °C for 6 h to remove any contaminants. Collected filters were individually stored in glass jars with a Teflon-lined screwed cap at − 20 °C to limit chemical reactions on the filter and losses of volatile compounds. Possible contamination from the ship exhaust was avoided by shutting off the sampling pump when the air came from the beam or when the relative wind speed was low (< 5 m s^−1^).

### Collection and analysis of bulk surface seawater samples

During the cruise, bulk surface seawater (SSW) samples were collected using a bucket during each aerosol sampling period^24,25,31^. It is noted that the SSA sampling, which was related to the SSW samples, covered the average distance of 145 ± 77 km moved by the R/V *Hakuho Maru*. For Chl *a* and Chllide *a* measurements, duplicate seawater samples with each of 0.5–1.2 L were collected at 0 m of each sampling station using a clean plastic bucket. The samples were filtered through 25 mm Whatman GF/F filters (nominal pore size of 0.7 µm) with a gentle vacuum (< 0.013 MPa). Each filter was blotted with filter paper, frozen in liquid nitrogen, and stored in an ultra-freezer (< − 70 °C) until analysis on land. Suzuki et al.^17^ described how phytoplankton pigments were extracted with a DMF-bead-beating technique and analyzed using ultra-high-performance liquid chromatography (UHPLC)^44^.

POC concentration was determined according to the method described by Nosaka et al.^18^. The seawater sample was filtered onto pre-combusted Whatman GF/F filters (25-mm in diameter, 450 °C for 5 h) under a gentle vacuum (< 0.013 MPa) and stored at − 20 °C until analysis. The samples were thawed at room temperature and exposed to hydrochloric acid (HCl) fumes to remove inorganic carbon, followed by complete drying in a vacuum desiccator for more than 24 h. The POC concentrations on the filters were determined using an online elemental analyzer (FlashEA1112, Thermo Fisher Scientific, Inc., USA).

DOC concentrations were measured according to a method described by Tanaka et al.^45^ The seawater samples were filtered with a 0.22-μm Durapore filter (Millipore) under gentle vacuum. The filtrate was transferred into a pre-combusted borosilicate glass vial with an acid-cleaned Teflon-lined cap^24^. The samples were then kept frozen at − 20 °C in the dark until analysis. DOC analysis was conducted using a total organic carbon (TOC) analyzer (Model TOC-V, Shimadzu).

### Chemical analysis of the submicrometer aerosols

In the present study, the term water-soluble aerosols are defined as particles sampled on the filter and extracted with ultrapure water followed by filtration through a syringe filter^31^. To determine the WSOC concentration of the submicrometer filter samples, another filter cut of 3.14 cm^2^ was extracted with 20 mL ultrapure water using an ultrasonic bath for 15 min. The extracts were filtered through a 0.22 µm pore syringe filter, before being injected into a TOC analyzer (Model TOC-L_CHP_, Shimadzu)^31^. Additionally, another cut of the filter (3.14 cm^2^) was extracted with 10 mL of ultrapure water under ultrasonication to determine the concentration of major inorganic ions, including Na^+^. The same syringe filter type was used, before the extract was injected into an ion chromatograph (Model 761 compact IC; Metrohm)^31^. The mass concentrations of OC and EC were measured using a Sunset Laboratory OC-EC analyzer. A filter punch of 1.54 cm^2^ was used for this analysis.

Another portion of the filter (3.80 cm^2^) was extracted with dichloromethane/methanol to measure glucose as a biogenic molecular tracer. The –OH functional groups in the extracts were reacted with N,O-bis-(trimethylsilyl) trifluoroacetamide (BSTFA) to form trimethylsilyl (TMS) ethers. The TMS derivatives were then analyzed using a capillary gas chromatograph (GC7890, Agilent) coupled to a mass spectrometer (MSD5975C, Agilent)^31^.

### Stable carbon isotopic characterization of the aerosols

For the determination of δ^13^C_WSOC_, another filter cut (14.14 cm^2^) from the same aerosol filter sample was acidified to pH 2 with HCl to remove inorganic carbon before extraction. The decarbonated filter samples were then dried under a nitrogen stream for approximately 2 h. WSOC was extracted from the filters in 20 mL of ultrapure water using the method described above to measure the WSOC concentration. The extracted samples were concentrated via rotary evaporation, and 40 μL of each sample was transferred to be absorbed onto 10 mg of pre-combusted Chromosorb in a pre-cleaned tin cup. The ^13^C_WSOC_ was then measured using an elemental analyzer (EA; NA 1500, Carlo Erba, Milan, Italy) interfaced with an isotope ratio mass spectrometer (IRMS; Finnigan MAT Delta Plus, Thermo Finnigan, San Jose, CA, USA). Also, the δ^13^C of total carbon (δ^13^C_TC_) (i.e., without water extraction) was measured with the EA–IRMS for the same aerosol filter samples^46^. The ^13^C data were reported relative to an established reference of carbon Vienna Pee Dee Belemnite (VPDB). Further details of the analytical method used for isotopic analysis are provided by Miyazaki et al.^22^.

### Offline measurement and determination of hygroscopicity *κ*

To measure hygroscopicity parameters of submicron water-soluble aerosols, a filter cut of 0.79 cm^2^ was extracted with 7 mL of ultrapure water using an ultrasonic bath (5 min × 3 times). The extracts were filtered through a 0.22 µm pore syringe filter (Millex-GV, Millipore) to remove any insoluble particles with diameters larger than 0.22 µm. Polydisperse aerosols were generated by first atomizing the filter extracts, followed by being dried at < 5% relative humidity with two diffusion dryers containing silica gel and molecular sieve in series. After passing the impactor and bipolar charger of the electrostatic classifier (TSI Model 3080), particles with a specific dry mobility diameter (D_dry_) of 45 nm were selected using a differential mobility analyzer (DMA, TSI Model 3081). That particle size is in the range of particles that activate as CCN at supersaturations lower than 1.0%, which are typically found in the ambient atmosphere^41,47^. The classified flow was then split into two parallel streams. The first went into the condensation particle counter (CPC, TSI Model 3775) to measure the total concentration of condensation nuclei (N_CN_). Whereas the other stream was channeled into a continuous-flow thermal-gradient diffusion chamber (CCN-100, Droplet Measurement Technologies) to measure the number concentration of CCN (N_CCN_)^48^.

N_CCN_ with a constant D_dry_ of 45 nm was measured at fifteen specific supersaturation (SS) levels between 0.25% and 0.8% in the CCN counter. Each SS was measured for 15 min, where N_CCN_ was obtained during the last 3 min when the SS was stable in the CCN chamber. The CCN activity of the particles generated was characterized by the critical supersaturation (SS_c_) at the specific D_dry_. The SS_c_ of CCN is defined as the SS at which N_CCN_ reaches 50% of the total N_CN_. The SS_c_ was determined by expressing the N_CCN_/N_CN_ ratio for SS and fitting the data to a sigmoid curve. The hygroscopicity parameter κ was calculated according to Petters and Kreidenweis^49^. Further details of the instrumental setup and the calculation of hygroscopicity *κ* are described elsewhere^48,50^.

The *κ* of OM (*κ*_OM_) was calculated by applying a mixing rule^49^ with the aerosol chemical composition in the SSA samples as follows:

2 $$\kappa =\varepsilon _{{{\text{SS}}}} \kappa_{{{\text{SS}}}} + \varepsilon_{{{\text{OM}}}} \kappa_{{{\text{OM}}}}$$

where *κ*_SS_ is a typical *κ* value for sea salt (~ 1.25)^38^. The *ε*_SS_ and *ε*_OM_ values are the volume fractions of sea salt and OM, respectively, which were calculated from the mass concentrations using the density of sea salt (ρ_SS_ = 1.8 g cm^−3^) and the assumed density of OM (ρ_OM_ = 1.4 g cm^−3^)^38^. Aerosol sea salt concentrations were calculated by 3.26 × [Na^+^], where 3.26 and [Na^+^] are the seawater ratio of (Na^+^ + Mg^2+^ + Ca^2+^ + K^+^ + Cl^−^ + SO_4_^2−^ + HCO_3_^−^)/Na^+^ and the mass concentration of Na^+^, respectively^13^.

## Supplementary information

Supplementary Figure S1.
