# Author Correction: New index of organic mass enrichment in sea spray aerosols linked with senescent status in marine phytoplankton

**DOI:** 10.1038/s41598-021-00643-6

**Published:** 2021-10-25

**Authors:** Yuzo Miyazaki, Koji Suzuki, Eri Tachibana, Youhei Yamashita, Astrid Müller, Kaori Kawana, Jun Nishioka

**Affiliations:** 1 Institute of Low Temperature Science, Hokkaido University, Sapporo, Japan; 2 Faculty of Environmental Earth Science, Hokkaido University, Sapporo, Japan; 3 Graduate School of Environmental Science, Hokkaido University, Sapporo, Japan; 4 Present Address: National Institute for Environmental Studies, Tsukuba, Japan; 5 Graduate School of Environmental Studies, Nagoya University, Nagoya, Japan; 6 Present Address: Japan Agency for Marine-Earth Science and Technology, Yokohama, Japan

Correction to: *Scientific Reports* 10.1038/s41598-020-73718-5, published online 12 October 2020

The original version of this Article contained errors in the Reference list.

The authors omitted the below References, which are now listed as References 46 and 48.

46. Kawamura, K., *et al*. Organic and inorganic compositions of marine aerosols from East Asia: Seasonal variations of water soluble dicarboxylic acids, major ions, total carbon and nitrogen, and stable C and N isotopic composition. In *Geochemical Investigations in Earth and Space Science: A Tribute to Isaac R. Kaplan,* Vol. 9 (eds Hill, R.J., *et al.*) 243–265. 10.1016/S18739881(04)80019-1 (The Geochemical Society, 2004).

48. Boreddy S. K. R. & Kawamura K.: Investigation on the hygroscopicity of oxalic acid and atmospherically relevant oxalate salts under sub- and supersaturated conditions. *Environ. Sci. Processes Impacts*, **20,** 106. 10.1039/c8em00053k (2018).

In addition, References 44 and 47 were provided in the Reference List but not cited in the body of the text.

As a result, in the Methods section, subheading ‘Collection and analysis of bulk surface sweater samples‘,

“Suzuki et al.^17^ described how phytoplankton pigments were extracted with a DMF-bead-beating technique and analyzed using ultra-high-performance liquid chromatography (UHPLC)^42^.”

now reads:

“Suzuki et al.^17^ described how phytoplankton pigments were extracted with a DMF-bead-beating technique and analyzed using ultra-high-performance liquid chromatography (UHPLC)^44^.”

Under the subheading ‘Stable carbon isotopic characterization of the aerosols‘,

“Also, the δ^13^C of total carbon (δ^13^C_TC_) (i.e., without water extraction) was measured with the EA–IRMS for the same aerosol filter samples.”

now reads:

“Also, the δ^13^C of total carbon (δ^13^C_TC_) (i.e., without water extraction) was measured with the EA–IRMS for the same aerosol filter samples^46^.”

Under the subheading ‘Offline measurement and determination of hygroscopicity *κ*‘,

“That particle size is in the range of particles that activate as CCN at supersaturations lower than 1.0%, which are typically found in the ambient atmosphere^41^.”

now reads:

“That particle size is in the range of particles that activate as CCN at supersaturations lower than 1.0%, which are typically found in the ambient atmosphere^41,47^.”

Under the same heading,

“Whereas the other stream was channeled into a continuous-flow thermal-gradient diffusion chamber (CCN-100, Droplet Measurement Technologies) to measure the number concentration of CCN (N_CCN_)^47^”.

now reads:

“Whereas the other stream was channeled into a continuous-flow thermal-gradient diffusion chamber (CCN-100, Droplet Measurement Technologies) to measure the number concentration of CCN (N_CCN_)^48^.”

And,

“Further details of the instrumental setup and the calculation of hygroscopicity *κ* are described by Müller et al.^47^.”

now reads:

“Further details of the instrumental setup and the calculation of hygroscopicity *κ* are described elsewhere^48,50^.”

As a result of the changes, References were renumbered accordingly.

Lastly, the Acknowledgements section was incomplete.

“This research was supported by Grants-in-Aid for Scientific Research (B) (25281002) from the Ministry of Education, Culture, Sports, Science and Technology (MEXT), Japan. M. Mochida and S. Kagami are acknowledged for their help with the atmospheric measurements. We thank K. Yoshida and A. Kamimura for their help in the measurements of POC and algal pigments, respectively. Thanks also go to H. Kawakami for her help with the chemical analysis of the aerosol samples. This study was also supported by the Grant for Joint Research Program of the Institute of Low Temperature Science, Hokkaido University.”

now reads:

“This research was supported by Grants-in-Aid for Scientific Research (B) (25281002) from the Ministry of Education, Culture, Sports, Science and Technology (MEXT), Japan. The authors deeply acknowledge K. Kawamura, who set up the EA-IRMS and the CCNC–SMPS systems that were used in this study to derive *k* values. M. Mochida and S. Kagami are acknowledged for their help with the atmospheric measurements. We thank K. Yoshida and A. Kamimura for their help in the measurements of POC and algal pigments, respectively. Thanks also go to H. Kawakami for her help with the chemical analysis of the aerosol samples. This study was also supported by the Grant for Joint Research Program of the Institute of Low Temperature Science, Hokkaido University.”

The original Article has been corrected.

