# Supplementary material for: New index of organic mass enrichment in sea spray aerosols linked with senescent status in marine phytoplankton
Source: Sci Rep. 2020 Oct 12;10:17042. doi: 10.1038/s41598-020-73718-5 (PMC7550598; doi:10.1038/s41598-020-73718-5)
Supplement: Supplementary file 1 — Supplementary Figure S1. [file 41598_2020_73718_MOESM1_ESM.pdf]

Supplementary Information for the manuscript:

New index of organic mass enrichment in sea spray aerosols linked with senescent status in marine phytoplankton

Yuzo Miyazaki[1], Koji Suzuki[2], Eri Tachibana[1], Youhei Yamashita[2], Astrid Müller[1,3,4], Kaori Kawana[5,6], and Jun Nishioka[1]

1 Institute of Low Temperature Science, Hokkaido University, Sapporo, Japan.

2 Faculty of Environmental Earth Science, Hokkaido University, Sapporo, Japan.

3 Graduate School of Environmental Science, Hokkaido University, Sapporo, Japan.

4 Now at National Institute for Environmental Studies, Tsukuba, Japan.

5 Graduate School of Environmental Studies, Nagoya University, Nagoya, Japan.

6 Now at Japan Agency for Marine-Earth Science and Technology, Kanagawa, Japan.

Correspondence and requests for materials should be addressed to Y. M. (email: yuzom@lowtem.hokudai.ac.jp)

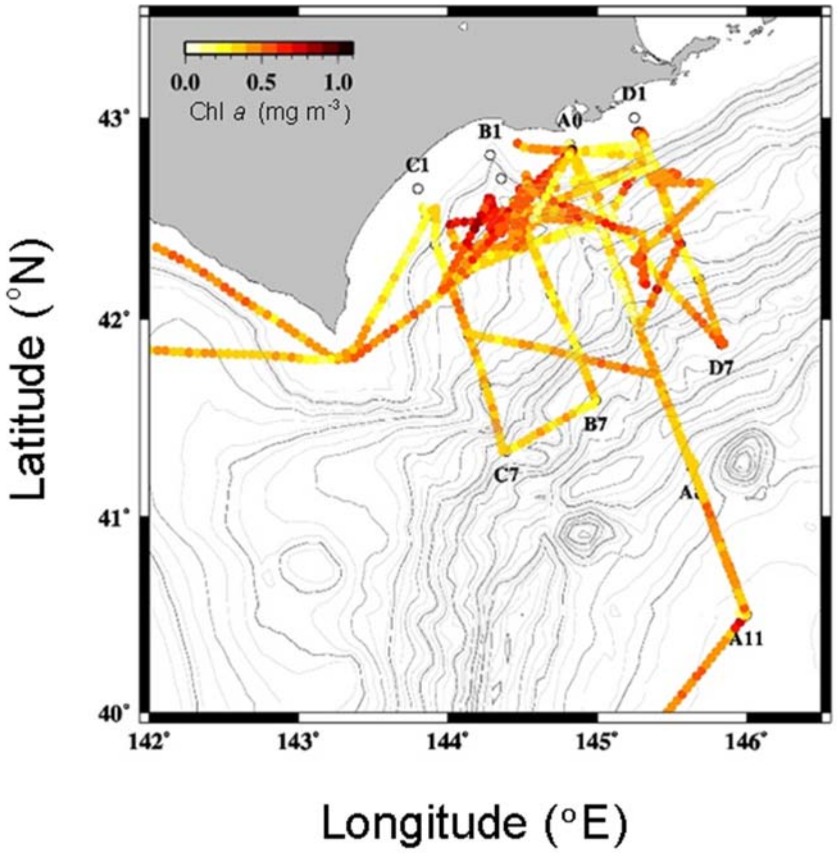

Figure S1. In vivo Chl *a* fluorescence concentrations at 5 m along the cruise track during the KH-15-1 expedition. The fluorescence levels were determined with a Chl *a* fluorometer (Model FLRT, WET Labs Inc.) of the surface monitoring system. The Generic Mapping Tools (GMT) 5 (https://www.generic-mapping-tools.org/) were used to create the figure.
